# Spatial-temporal analysis of hepatitis B in Fujian Province, China in 2012–2021

**DOI:** 10.1016/j.imj.2024.100110

**Published:** 2024-04-19

**Authors:** Shuo Yin, Shenggen Wu, Jingru Huang, Shutong Ren, Weijiang Xie, Xian'e Peng

**Affiliations:** a Department of Epidemiology and Health Statistics, School of Public Health, Fujian Medical University, Fuzhou 350122, China; b Fujian Provincial Center for Disease Control and Prevention, Fuzhou 350122, China; c College of Integrated Chinese and Western Medicine, Fujian University of Traditional Chinese Medicine, Fuzhou 350108, China; d Key Laboratory of Gastrointestinal Cancer (Fujian Medical University), Ministry of Education, School of Basic Medical Sciences, Fujian Medical University, Fuzhou 350108, China

**Keywords:** Hepatitis B, Viral hepatitis, Spatial epidemiology, Epidemic characteristics

## Abstract

**Background:**

Fujian Province has one of the highest reported incidences of hepatitis B virus infection in China. This study aimed to provide a theoretical framework for preventing and controlling hepatitis B in Fujian Province, and to assess the trends and the spatial-temporal distribution patterns of hepatitis B in this region.

**Methods:**

Data on hepatitis B cases were extracted from the National Notifiable Infectious Disease Surveillance System. Spatial autocorrelation analysis, trend surface analysis, and spatial-temporal scanning statistics were used to identify the spatial and aggregation patterns at the county level. The Joinpoint was used to assess the reported incidence trends.

**Results:**

The average reported incidence of hepatitis B in Fujian from 2012 to 2021 was 14.46/10,000 population, with 583,262 notified cases. The age-adjusted reported incidence of hepatitis B decreased from 17.44/10,000 population in 2012 to 11.88/10,000 population in 2021, with an average reduction in the annual percentage change of 4.5%. There were obvious spatial-temporal aggregation characteristics in hepatitis B cases, and a high-incidence area was located in eastern Fujian. Spatio-temporal scanning statistics revealed four levels of aggregation of hepatitis B reporting rates. The first level of aggregation area included Minhou, Gulou, Jin'an, Taijiang, and nine other districts and counties.

**Conclusion:**

The incidence of hepatitis B is declining in Fujian Province. Spatial clusters of hepatitis B cases in Fujian Province were identified, and high-risk areas in eastern Fujian still exist. Closely monitoring the general patterns in the occurrence of hepatitis B and implementing focused control and preventative strategies are important.

## Highlights

• Our study described the characteristics distribution of hepatitis B incidence.
• Our study includes data on the incidence of hepatitis B over the past 10 years.
• The reported incidence of hepatitis B in Fujian Province is declining.
• Our study provides a basis for allocating healthcare resources.

## Graphical abstract

**Figure:**
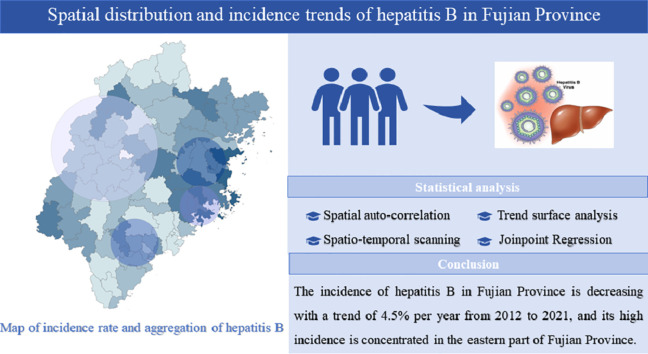

## 1 Introduction

Hepatitis B is an acute or chronic liver infection caused by the hepatitis B virus (HBV) [1]. While chronic hepatitis B can cause liver disease and potentially develop into cirrhosis and liver cancer, acute hepatitis B typically resolves on its own [2,3]. Liver diseases caused by HBV are the seventh leading cause of death worldwide [4]. According to the World Health Organization, approximately 296 million people worldwide have chronic hepatitis B, and approximately 1.5 million people are infected each year [5]. In China, approximately 70 million people are infected with HBV, 40% of whom have chronic hepatitis B, which leads to a heavy financial burden on individuals, families, and society. The World Health Assembly adopted the global health sector plan in 2016 in response to the danger to public health. The strategy of this plan aims to eradicate viral hepatitis by 2030 and reduce new infections by 90% and mortality by 65% [6]. The Lancet Gastroenterology & Hepatology Commission also launched an initiative to accelerate the elimination of viral hepatitis, calling for the measurement of national progress towards viral hepatitis elimination [7]. Key policies must be developed to accomplish these objectives. However, the creation and execution of new HBV regulations have been slow, posing a growing problem for nations such as China that are severely affected by HBV.

Previous studies have shown that there is a regional aggregation in the occurrence of hepatitis B [8], [9], [10], and the reported incidence varies greatly across regions. Therefore, the only way to effectively reduce the incidence of hepatitis B and maximize cost-effectiveness is to identify priority strategies based on characteristics of the local disease epidemic and implement targeted hepatitis B prevention and control measures at the provincial level. Spatial epidemiology is a branch of epidemiology that can effectively reveal the spatial distribution patterns of diseases [11,12], examine the influencing factors of diseases [13], and provide a basis for disease prevention and health resource allocation [14,15]. Hepatitis B high-risk regions in China are primarily found in the southeast and northwest of this nation [16]. Fujian Province, which is located in the southeastern coastal area, has a particularly high prevalence of hepatitis B, with the fifth highest reported incidence in China from 1990 to 2017 [17]. However, existing research on the spatial distribution of the hepatitis B incidence in various regions of Fujian Province is limited. This study aimed to determine the trend and distribution pattern of hepatitis B in Fujian Province from 2012 to 2021 to provide information on the prevention and control of hepatitis B and to provide some basis for the appropriate distribution of medical and health resources.

## 2 Materials and methods

### 2.1 Data source

**Fig. 1:**
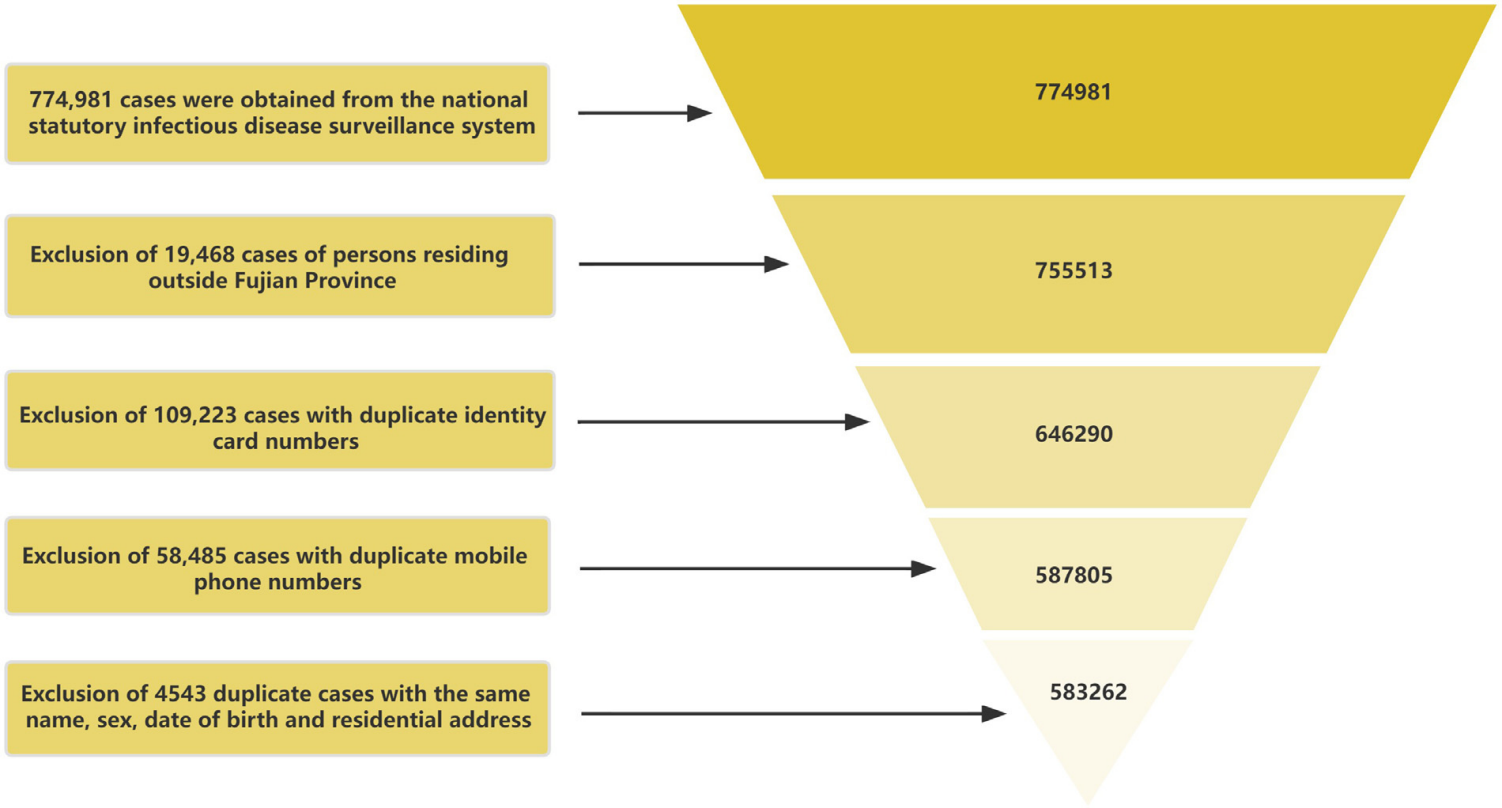
Flowchart of screening data of reported cases of hepatitis B in Fujian Province.

Data on hepatitis B were obtained from the China Information System for Disease Control and Prevention, which is a web-based infectious disease reporting system that was established in 2004 covering 40 notifiable diseases. According to the requirements of the China Information System for Disease Control and Prevention, medical institutions and disease prevention and control institutions at all levels nationwide must report to this system in the first instance when the above-mentioned 40 notifiable diseases are detected. A total of 774,981 reported cases of hepatitis B were collected from 2012 to 2021. Some of these cases were duplicated because of multiple patient admissions. Therefore, we used Microsoft Office Excel 2016 software to retain the data of the earliest reported cases by flagging and deleting information with the same name, sex, identity card number, and mobile phone number. Finally, 583,262 cases were included in this study (Fig. 1).

Demographic data (year-end resident population) were obtained from the Fujian Provincial Bureau of Statistics (https://tjj.fujian.gov.cn/xxgk/njgb/tjnj/). The administrative map of Fujian Province was from the National Geographics Center of China.

### 2.2 Temporal trend analysis

A temporal trend analysis was conducted using the Joinpoint method. We used the Joinpoint Regression Program (Version 5.0.2. May 2023) from the National Cancer Institute Surveillance and Research Program to build a connection point regression model (log-linear model) to reflect the long-term trend of the reported incidence of hepatitis B. Measurement of the direction and magnitude of trends over time was performed by calculating the annual percentage change (APC), average annual percentage change (AAPC), and associated 95% confidence interval (CI). A two-sided *P* value < 0.05 was considered statistically significant [18].

### 2.3 Spatial autocorrelation analysis and trend surface analysis

Spatial autocorrelation analysis is a spatial statistical method that can reveal the regional structure of spatial variables and verify whether an element attribute value is associated with an attribute value at an adjacent space point. This mainly includes global autocorrelation analysis and local autocorrelation analysis. Trend surface analysis is a statistical method for investigating the spatial distribution and local variation of a disease by fitting a linear model to sample data using a mathematical surface and building a binary polynomial regression model.

Global Moran's *I* values and trend surface maps derived from ArcGIS 10.8 software (ESRI Inc. Redlands, CA, USA) were used to identify spatial autocorrelation and trends in the reported incidence of hepatitis B in Fujian Province. Global Moran's *I* was used as an indicator to reflect global spatial aggregation in the whole region, and a significance test was performed. Moran's *I* index ranges approximately from –1 to 1. An index < 0 indicates a negative correlation, 0 indicates no correlation, and > 0 indicates a positive correlation. Anselin Local Moran's *I* was used to evaluate the correlation of the same attributes between each spatial unit and neighboring units and determine the “high-high” (clusters of cities with a high reported incidence), “low-low” (clusters of cities with a low reported incidence), “low-high” (cities with a low reported incidence that are surrounded mainly by cities with a high reported incidence), and “high-low” (cities with a high reported incidence that are surrounded mainly by cities with a low reported incidence) distributions of the spatial distribution.

### 2.4 Spatial and temporal scanning statistics

Retrospective space-time cluster analysis using the space-time permutation statistic was employed for the reported incidence of hepatitis B in 83 counties and districts of Fujian Province from 2012 to 2021. SaTScan (Version 10.1, July 2022) software was developed by Martin Kulldorff together with Information Management Services Inc. and can be downloaded on the Web (https://www.satscan.org/). This software is used to analyze spatial, temporal, and spatial-temporal data using spatial, temporal, or spatial-temporal scanning statistics. The method of this analysis is based on a moving column scanning window, with the base of the column corresponding to the geographic region and the height of the column corresponding to time. In each scanning window, the theoretical incidence can be calculated according to the total incidence and the number of people in the window. The logarithmic likelihood ratio (LLR) is then constructed by using the actual incidence and theoretical incidence inside and outside the scanning window, respectively. The window with the largest LLR, which is the window with the strongest clustering, is selected from all of the scanning windows. The secondary clusters are the other windows with statistically significant LLR values. The maximum spatial scanning area was set as 50% of the total population of the province, scanned in “years,” and the period was from January 2012 to December 2016. The Monte Carlo simulation test was used to evaluate whether the difference was statistically significant. The number of simulations was equal to 999 [19]. ArcMap 10.8 software was used to visualize the scanning results.

## 3 Results

### 3.1 Overall trend of the reported incidence of hepatitis B in Fujian Province

The demographic details of hepatitis B cases are shown in Table 1. There were 583,262 reported cases of hepatitis B included in this study, of which 67.37% were men and 65.45% were individuals aged between 20 and 50 years old. The reported incidence of hepatitis B in Fujian Province has decreased over the past 10 years, from 18.31/10,000 people in 2012 to 11.92/10,000 people in 2021.

**Table 1:** Population characteristics of hepatitis B in Fujian Province.

	2012	2013	2014	2015	2016	2017	2018	2019	2020	2021	Total
Gender											
Male	49299	48524	45291	42240	37997	33866	33811	36209	33280	32454	392971
Female	21041	22022	21212	20327	18357	17087	16926	18423	17445	17451	190291
Sex ratio	2.34	2.20	2.14	2.08	2.07	1.98	2.00	1.97	1.91	1.86	2.07
Age											
0–9	312	306	275	251	246	199	204	170	144	142	2249
10–19	2544	2125	1561	1298	1087	755	629	615	466	453	11533
20–29	20361	18979	16676	14410	12087	9933	8767	8186	6155	5035	120589
30–39	15626	15850	16210	13587	12560	11439	11408	13216	12138	13236	135270
40–49	13856	14398	12694	13804	12244	11572	11710	12641	11734	11239	125892
50–59	9306	10040	10056	9992	9372	8695	8973	10129	10290	10690	97543
60–69	5393	5809	5981	6335	5881	5665	6267	6518	6540	5981	60370
70–79	2349	2441	2403	2289	2286	2090	2168	2421	2571	2464	23482
80–99	593	598	647	601	591	605	611	736	687	665	6334
Number of reported cases	70340	70546	66503	62567	56354	50953	50737	54632	50725	49905	583262
Total population (million)	38.41	38.85	39.45	39.84	40.16	40.65	41.04	41.37	41.61	41.87	403.25
Reported incidence (per 10000 population)	18.31	18.16	16.86	15.70	14.03	12.53	12.36	13.21	12.19	11.92	14.46

**Fig. 2:**
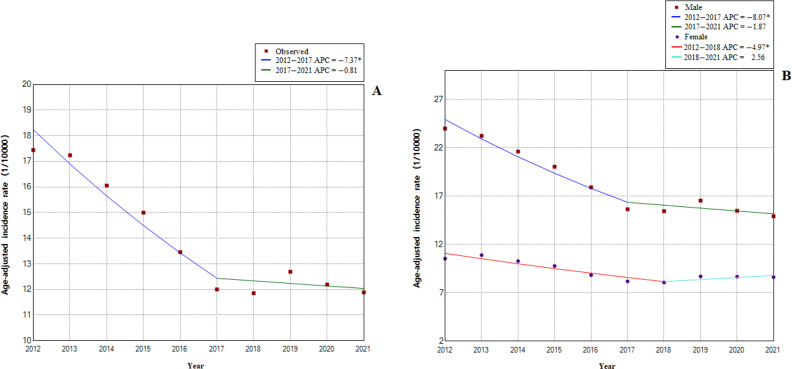
Joinpoint model estimates in Fujian from 2012 to 2021. (A) Trends in the overall reported incidence of hepatitis B; (B) trends in the reported incidence of hepatitis B stratified by sex.

Joinpoint regression showed that the long-term trends in the APC of the total population age-adjusted reported incidence of hepatitis C could be divided into two stages (Fig. 2A). From 2012 to 2017, the age-adjusted reported incidence of hepatitis B significantly decreased from 17.44/10,000 population in 2012 to 12.00/10,000 population in 2017. The APC decreased by 7.4% (95% CI, −9.8% to −4.9%; *P* = 0.001). From 2017 to 2021, the age-adjusted reported incidence of hepatitis B decreased from 12.00/10,000 population in 2017 to 11.88/10,000 population in 2021. Additionally, the APC decreased by 0.8% (95% CI, −4.8% to 3.4%; *P* = 0.636). The AAPC decreased by 4.5% (95% CI, −6.2% to −2.8%; *P* < 0.001).

There were two distinct phases to the long-term trends of the male age-adjusted reported incidence of hepatitis B (Fig. 2B). From 2012 to 2017, the APC decreased by 8.1% (95% CI, −10.5% to −5.6%; *P* < 0.001), and from 2017 to 2021, it decreased by 1.9% (95% CI −6.0% to 2.4%; *P* = 0.307). The AAPC of the male age-adjusted reported incidence decreased by 5.4% (95% CI, −7.1% to −3.6%; *P* < 0.001). The APC for the female age-adjusted reported incidence decreased by 5.0% (95% CI, −7.3% to −2.6%; *P* = 0.003) from 2012 to 2018 and increased by 2.6% (95% CI, −4.6% to 10.2%; *P* = 0.407) from 2018 to 2021. The AAPC of the female age-adjusted reported incidence decreased by 2.5% (95% CI, −4.7% to −0.3%; *P* = 0.023) from 2012 to 2021.

### 3.2 Spatial distribution and spatial autocorrelation analysis of the reported incidence of hepatitis B in Fujian Province

**Fig. 3:**
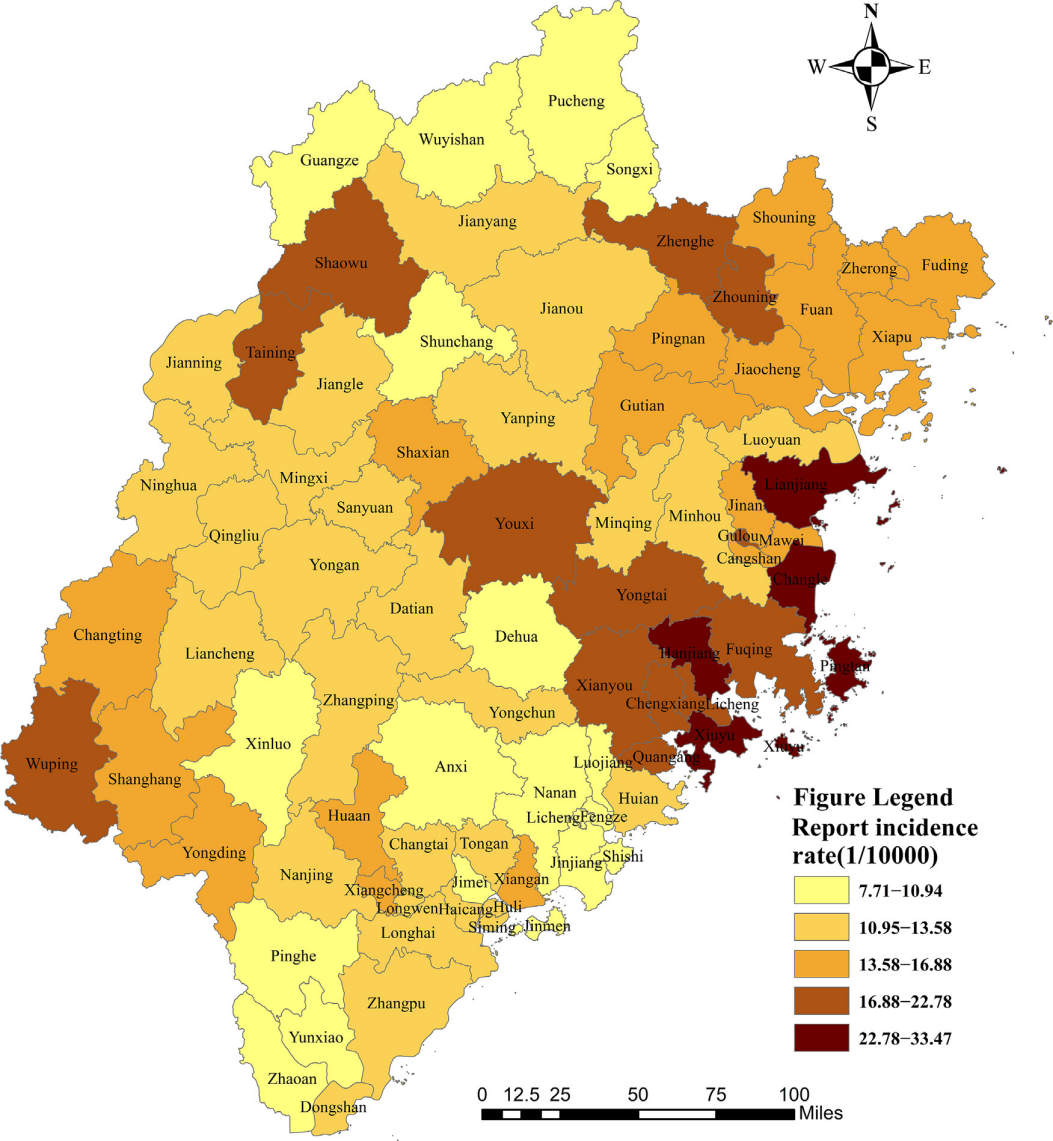
The average reported incidence of hepatitis B in each district/county of Fujian Province, China. Note: Produced on the Ministry of Natural Resources Standard Map Service website GS(2022) 1873, with no modifications to the base map boundaries.

The average reported prevalence of hepatitis B by county and district in Fujian Province between 2012 and 2021 is shown in Fig. 3. In the spatial distribution vector map, the reported incidence of hepatitis B showed a trend of spatial aggregation. The areas with a higher reported incidence were mainly situated in the coastal areas of Fujian, namely Xiuyu, Pingtan, Changle, Lianjiang, Hanjiang, and Gulou. In contrast, the areas with a lower reported incidence were situated in the north and southeast of Fujian, namely Zhaoan, Pucheng, Wuyishan, and Pinghe.

**Fig. 4:**
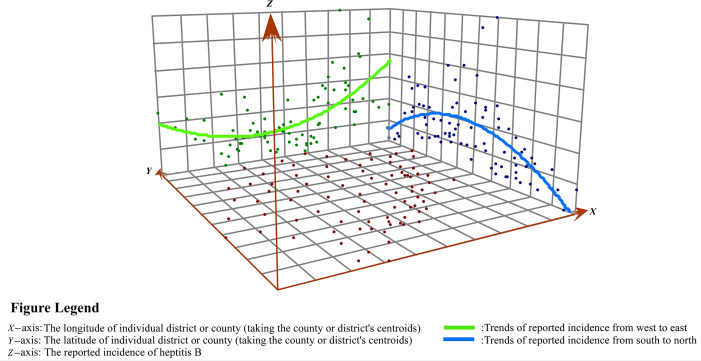
Trend surface analysis of hepatitis B reported incidence.

The trend analysis showed that the overall distribution of the reported incidence of hepatitis B in Fujian Province from 2012 to 2021 was U-shaped in the east-west direction, while an inverted U-shape was observed in the north-south direction (Fig. 4).

Table 2 shows the global spatial autocorrelation results of the reported incidence of hepatitis B in Fujian Province from 2012 to 2021. The global Moran's *I* ranged from 0.18 to 0.34 (*P*_each_ < 0.001), which indicated that the reported incidence of hepatitis B showed a spatial aggregation trend.

**Table 2:** Global spatial autocorrelation analysis of hepatitis B in Fujian Province, China in 2012–2021.

Year	Moran's *I*	*Z*-score	*P*-value
2012	0.30	4.55	<0.001
2013	0.24	3.70	<0.001
2014	0.28	4.28	<0.001
2015	0.23	3.57	<0.001
2016	0.23	3.63	<0.001
2017	0.26	3.99	<0.001
2018	0.25	3.90	<0.001
2019	0.34	5.16	<0.001
2020	0.20	3.20	<0.001
2021	0.18	2.80	<0.001
Average	0.26	4.00	<0.001

**Fig. 5:**
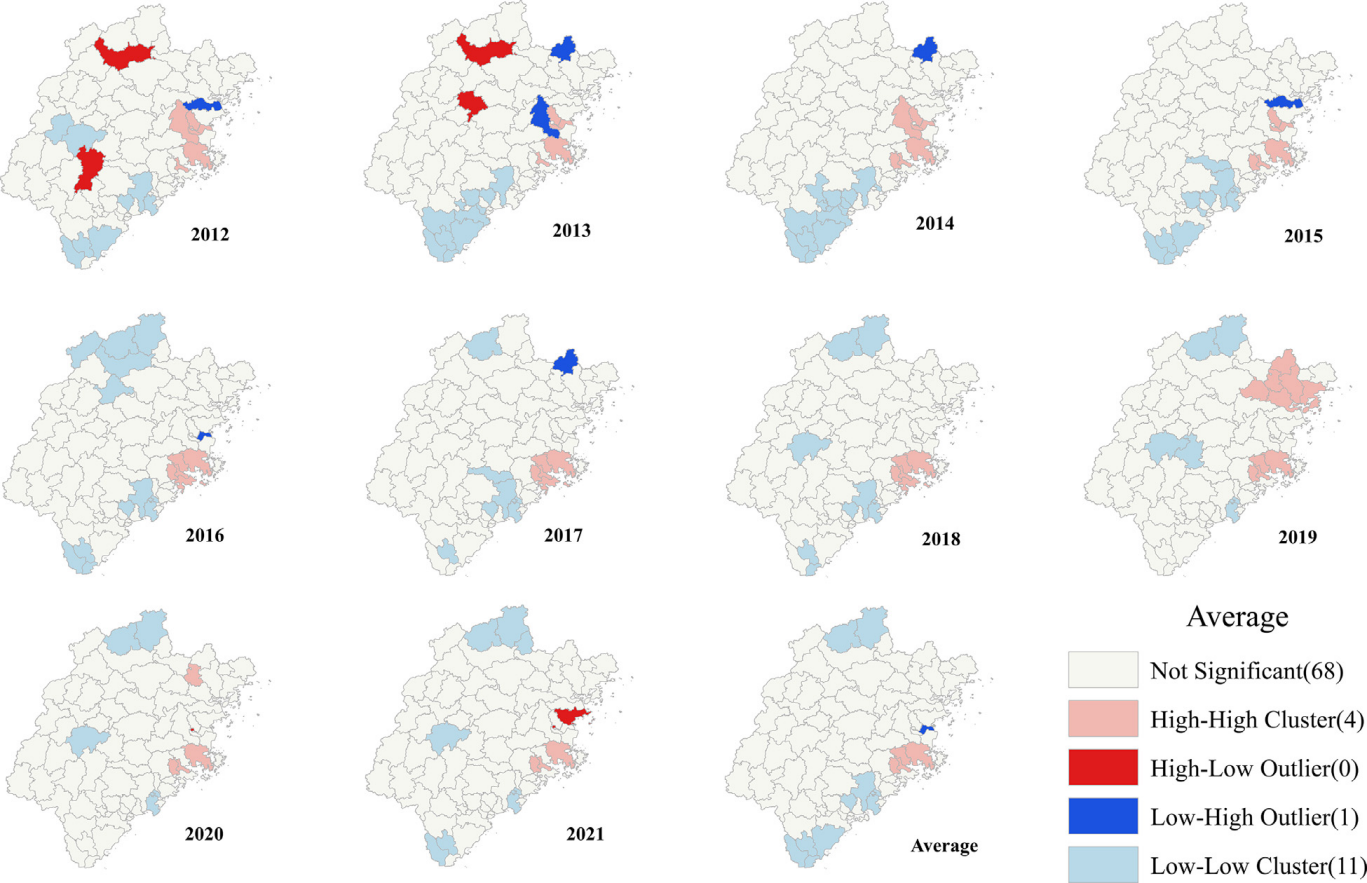
Spatial clusters of the reported incidence of patients with hepatitis B in Fujian Province, China.

The results of the global Moran's *I* index focused on the whole and ignored local features. Therefore, we needed to calculate the local aggregation of hepatitis B and analyze the regional specificity. Local Moran statistics were used to observe local aggregation of the reported incidence of hepatitis B. We found that the reported incidence of hepatitis B showed high or low-intensity spatial aggregation in local regions (Fig. 5). The average reported incidence of hepatitis B in the past 10 years showed that there were four counties in the high-high cluster areas and 11 counties in the low-low cluster areas. The high-high clustering areas were mainly in the east of Fujian Province, such as Cangshan, Licheng, Fuqing, and Hanjiang. The low-low clustering areas were situated in the north and south of Fujian Province, namely Nan'an, Yunxiao, Fengze, Jinjiang, Wuyishan, Zhao'an, and Dongshan. The low-high aggregation areas were Mawei, Minhou, and Shouning.

### 3.3 Distribution of the reported incidence of hepatitis B spatial-temporal clustering

**Fig. 6:**
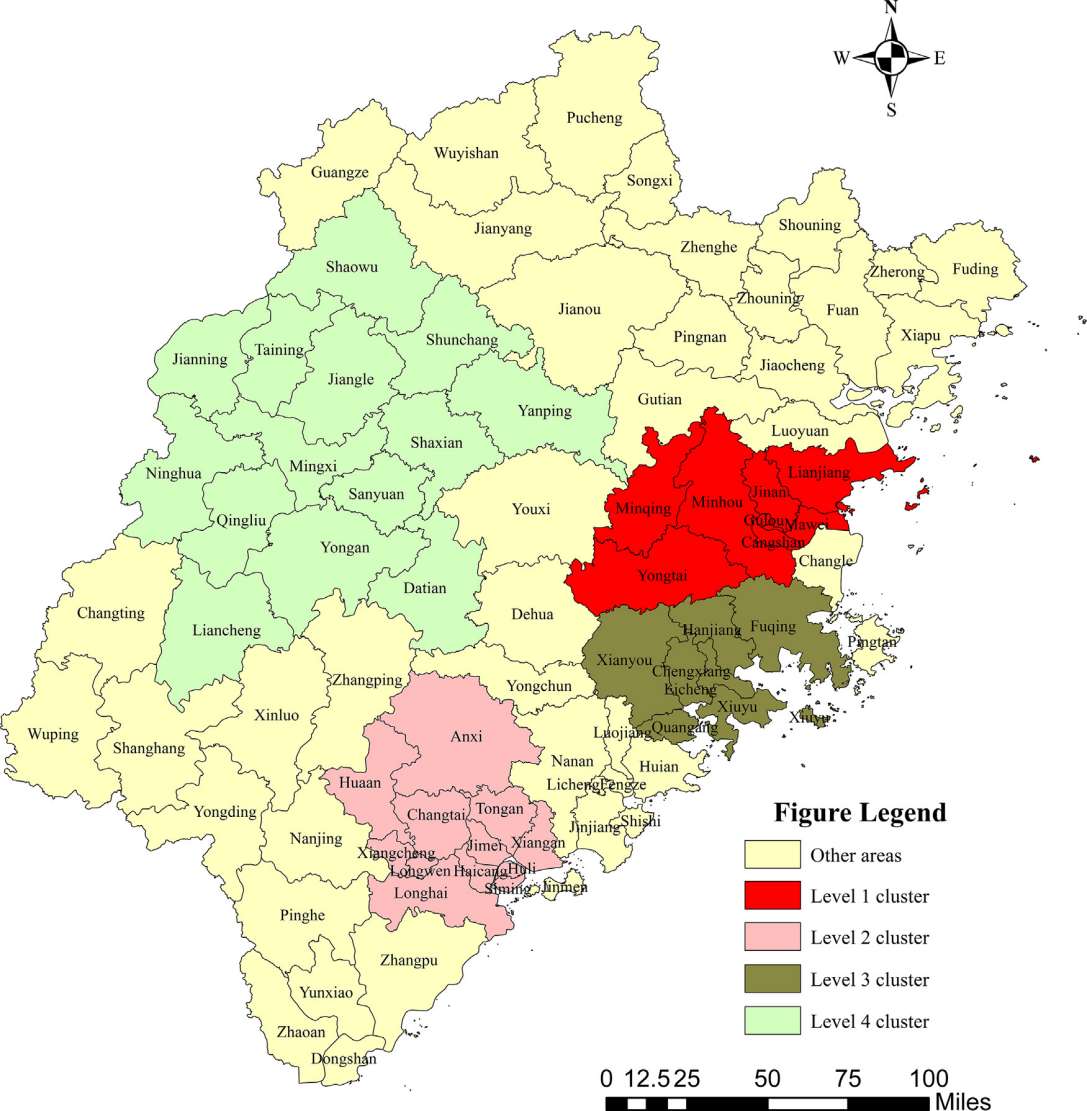
Distribution of spatial-temporal clustering of the reported incidence of hepatitis B in Fujian Province, China. Note: Produced on the Ministry of Natural Resources Standard Map Service website GS(2022) 1873, with no modifications to the base map boundaries.

From 2012 to 2021, four clusters were examined across Fujian Province (Table 3 and Fig. 6) The Level 1 cluster included nine districts or counties: Minhou, Gulou, Jin'an, Taijiang, Cangshan, Minqing, Mawei, Yongtai, and Lianjiang. The clustering time was from 2012 to 2015, mainly in eastern Fujian Province (RR = 1.24, LLR = 1145.9, *P* < 0.001).

**Table 3:** Spatiotemporal clusters of hepatitis B in Fujian Province, China in 2012–2021.

Most likely cluster	Cluster time frame	Coordinates/Radius	Observed cases	Expected cases	RR	LLR	*P*-value
Level 1 cluster	2012–2015	(26°17′74″N, 119°11′30″E) / 49.15 km	50897	41209	1.24	1145.9	<0.001
Level 2 cluster	2019–2021	(24°74′06″N, 117°80′00″E) / 48.16 km	32483	25591	1.27	897.1	<0.001
Level 3 cluster	2017–2021	(25°40′24″N, 119°08′30″E) / 39.60 km	49053	44882	1.09	204.31	<0.001
Level 4 cluster	2017–2018	(26°41′78″N, 117°20′60″E) / 104.84 km	8732	7717	1.13	64.86	<0.001

The Level 2 cluster mainly included 12 districts and counties (i.e., Changtai, Longwen, Xiangcheng, Jimei, Haicang, Tong'an, Hua'an, Longhai, Huli, Anxi, Siming, and Xiang'an). The clustering time was from 2019 to 2021, mainly in southern Fujian Province (RR = 1.27, LLR = 897.1, *P* < 0.001).

The Level 3 cluster mainly included seven districts and counties: Licheng, Chengxiang, Xiuyu, Hanjiang, Quangang, Fuqing, and Xianyou. The clustering time was from 2017 to 2021, mainly in eastern Fujian Province (RR = 1.09, LLR = 204.3, *P* < 0.001).

The Level 4 cluster mainly included 14 districts and counties (i.e., Mingxi, Jiangle, Sanyuan, Qingliu, Taining, Ninghua, Yong'an, Shaxian, Jianning, Shunchang, Datian, Shaowu, Liancheng, and Yanping). The clustering time was from 2017 to 2018, mainly in eastern Fujian Province (RR = 1.13, LLR = 64.9, *P* < 0.001).

## 4 Discussion

Infection with HBV is still a serious public health issue in China. Fujian is one of the provinces with the highest reported incidence of HBV in South China. However, no previous studies have focused on the spatial-temporal patterns for HBV in this area. To the best of our knowledge, our analysis covers the longest period and largest sample size studied in Fujian, mainland China. The average reported incidence of hepatitis B in Fujian Province decreased by 4.5% overall between 2012 and 2021, which is a major step towards the goal of eliminating hepatitis in China by 2030. In our study, we described the epidemiological features and trends of hepatitis B from 2012 to 2021.

The reported incidence of hepatitis B in this study showed significant declining trends from 2012 to 2021, which is consistent with previous studies. The following factors may have caused these patterns. First, universal hepatitis B immunization effectively reduced the incidence of hepatitis B. In China, the hepatitis B vaccine has been included in the newborn immunization program since 1992, although at that time, parents could be charged for the cost of hepatitis B vaccine and its administration [20]. In 2002, the hepatitis B vaccine was fully integrated into the routine infant immunization schedule, and it was fully free of charge in 2007 [21,22]. To date hepatitis B vaccination (three-dose, 0-1-6-month schedule) is listed in class A vaccines of the national immunization program. The coverage rates of birth-dose and three-dose hepatitis B vaccine are higher than 90% [23]. In addition, medical professionals, patients on hemodialysis, intravenous drug users, college students, and other high-risk groups for HBV infection are eligible for free hepatitis B vaccinations in China. The implementation of this series of policies played an essential role in alleviating the heavy burden of viral hepatitis B. Second, the World Health Organization developed ambitious targets for the elimination of HBV and hepatitis C virus as public health threats by 2030. China has actively participated in this program for the past 20 years. Additionally, by expanding national investment in immunization and health education initiatives, China has made remarkable progress toward preventing hepatitis [24]. An example of this progress is that China launched a major special national science and technology project to prevent and control viral hepatitis in 2009 [7,25,26]. This project effectively increased the coverage of hepatitis B vaccine in poverty-affected areas and closed the gap between eastern and western regions and between the rich and the poor. Finally, the diagnostic techniques for hepatitis B have become progressively well-rounded [27], making diagnosing and reducing the risk of viral transmission easy. These efforts led to a decrease in cases of all types of viral hepatitis, including hepatitis B.

The World Health Organization's 2030 target of eliminating viral hepatitis is still a long way in the future, despite the observed prevalence of hepatitis B in Fujian appearing to be significantly declining (decreasing the number of new cases to 0.2/10,000 by 2030). The government should guide the allocation of public health resources and influence vaccination strategies according to regional-specific conditions. Further efforts are required to increase vaccine coverage in high-risk rural regions with lower economic development and medical resource levels, and make policies for HBV blood screening in areas with a higher incidence.

There are sex disparities in the prevalence of hepatitis B. In line with other studies, in this study, the incidence of hepatitis B in men was higher than that in women [28]. An explanation for this finding could be that sex hormones, such as estrogen and testosterone, affect men's susceptibility to HBV infection [29,30]. In addition, men are more likely to develop poor lifestyle habits (such as smoking, drug abuse, and drinking alcohol) [31] than women, which may lead to an increased risk of HBV infection [32]. Furthermore, in the context of growing population mobility and increasingly open attitudes towards sex, men who have sex with men or female sex workers may also have an increased risk of hepatitis infection [33,34]. Regarding the distribution of age in this study, a high number of hepatitis B cases (65.45%) were reported by individuals aged 20–50 years, suggesting that hepatitis B has a large effect on labor productivity. Despite the fact that newborn hepatitis B vaccination has been listed in class A vaccines of the national immunization program [23], there is no united strategy, plan, or medical insurance for adults and adolescents aged older than 14 years who have not been vaccinated against hepatitis B [35]. This lack has resulted in low hepatitis B vaccination rates among adults [35]. Therefore, to lower the new incidence of hepatitis B, the government should broaden the area of hepatitis B vaccine immunization by concentrating on men who have sex with men, female sex workers, and other high-risk adult populations with HPV infection, and implement targeted prevention and control efforts.

In this study, local regional trends showed apparent heterogeneity, despite the overall declining temporal trend of the relative risk of HBV. According to the 10-year surveillance data, we found that the average reported incidence of hepatitis B showed an overall U-shaped distribution in the east-west direction and an inverted U-shaped distribution in the north-south direction. The areas of a higher incidence of hepatitis B were concentrated in the eastern coastal areas. Similarly, the local Moran statistical analysis showed that the high-high clustering areas were mainly in the eastern coastal areas of Fujian. Since 1978, China has embarked on a policy of internal reform and opening up to the outside world, a large number of rural surplus laborers have flocked to these coastal economically developed areas [36,37]. The majority of these laborers worked as fisherman or construction laborers. Farmers in China [16] are the primary population affected by hepatitis B because of their lower educational attainment and lack of understanding about health and immunization [38,39].

Most of these floating populations are young or middle-aged and in an active period of social activities and sexual behavior, which may increase the risk of HBV infection. The hepatitis B epidemic in Fujian Province from 2012 to 2021 clearly showed temporal and spatial clustering, with the highest incidence primarily centered in this province's eastern regions according to the results of the spatial-temporal scans. Four clusters were scanned, and the reported incidence of hepatitis B in the corresponding areas was at its peak during the clustering time. Level 1 clusters were centered in Minhou from 2012 to 2015, but these areas became non-aggregated as the reported incidence of hepatitis B decreased. Levels 2 and 3 clusters were still aggregated in 2021, suggesting that there is still a serious problem with hepatitis B in these locations. These results are different from those of the local autocorrelations because the spatial-temporal analysis examined the incidence of hepatitis B in the temporal and spatial dimensions and characterized the regional clustering of this disease in different time intervals. The high frequency in coastal areas should serve as a warning to the government to step up routine immunization programs in these areas and focus more on inspecting and identifying the high-risk population with HBV infection.

This study has some limitations. The Centres for Disease Control and Prevention information system is the source of the reported incidence statistics. Additionally, there is some reporting bias as a result of various local policies, the occurrence of duplicate reporting, and disparities in regional surveillance illness reporting capacities [40]. Moreover, this study only examined the development and distribution of the reported incidence of hepatitis B and did not analyze its influencing factors. Further efforts are required to investigate the influencing factors of the reported incidence of hepatitis B in Fujian Province, such as occupation, population density, medical level, and economic level. Additionally, HBV infection is greatly affected by individual activities, such as sexual activity, intravenous drug use, occupational exposure, and household contact [41,42]. This study is a study conducted at the county level, not at the individual level, and therefore may raise the issue of ecological fallacy [43]. More micro-factors need to be taken into account in the statistical analysis in future studies.

## 5 Conclusion

Although the reported incidence of hepatitis B has generally declined by 4.5% from 2012 to 2021, the prevention and control of hepatitis B in Fujian Province still need to be improved. Men and adults aged 20–50 years are high-risk populations for HBV infection, and the high-incidence areas are concentrated in the eastern part of Fujian Province. In high-risk areas and people, more efforts are required to improve vaccination coverage for HBV.

## Funding

This work was supported by the Fujian Research and Training Grants for Young and Middle-aged Leaders in Healthcare.
